# A novel intracellular antibody against the E6 oncoprotein impairs growth of human papillomavirus 16-positive tumor cells in mouse models

**DOI:** 10.18632/oncotarget.6925

**Published:** 2016-01-15

**Authors:** Carla Amici, Michela Visintin, Francesca Verachi, Francesca Paolini, Zulema Percario, Paola Di Bonito, Angela Mandarino, Elisabetta Affabris, Aldo Venuti, Luisa Accardi

**Affiliations:** ^1^ University of Rome Tor Vergata, Department of Biology, Via della Ricerca Scientifica, Rome, Italy; ^2^ Rottapharm Biotech Srl, Biotherapeutics Discovery, Area Science Park – Basovizza, Trieste, Italy; ^3^ Istituto Superiore di Sanità, Department of Infectious, Parasitic and Immunomediated Diseases, Viale Regina Elena, Rome, Italy; ^4^ Regina Elena National Cancer Institute, Laboratory of Virology and HPV-Unit, via delle Messi d'Oro, Rome, Italy; ^5^ University Roma Tre, Department of Science, Viale G. Marconi, Rome, Italy

**Keywords:** E6 oncoprotein, human papillomaviruses, cancer therapy, scFv, intracellular antibodies

## Abstract

Single-chain variable fragments (scFvs) expressed as “intracellular antibodies” (intrabodies) can target intracellular antigens to hamper their function efficaciously and specifically. Here we use an intrabody targeting the E6 oncoprotein of Human papillomavirus 16 (HPV16) to address the issue of a non-invasive therapy for HPV cancer patients.

A scFv against the HPV16 E6 was selected by Intracellular Antibody Capture Technology and expressed as I7nuc in the nucleus of HPV16-positive SiHa, HPV-negative C33A and 293T cells. Colocalization of I7nuc and recombinant E6 was observed in different cell compartments, obtaining evidence of E6 delocalization ascribable to I7nuc. In SiHa cells, I7nuc expressed by pLNCX retroviral vector was able to partially inhibit degradation of the main E6 target p53, and induced p53 accumulation in nucleus. When analyzing *in vitro* activity on cell proliferation and survival, I7nuc was able to decrease growth inducing late apoptosis and necrosis of SiHa cells.

Finally, I7nuc antitumor activity was demonstrated in two pre-clinical models of HPV tumors. C57BL/6 mice were injected subcutaneously with HPV16-positive TC-1 or C3 tumor cells, infected with pLNCX retroviral vector expressing or non-expressing I7nuc. All the mice injected with I7nuc-expressing cells showed a clear delay in tumor onset; 60% and 40% of mice receiving TC-1 and C3 cells, respectively, remained tumor-free for 17 weeks of follow-up, whereas 100% of the controls were tumor-bearing 20 days post-inoculum. Our data support the therapeutic potential of E6-targeted I7nuc against HPV tumors.

## INTRODUCTION

Antibodies in single-chain format (scFvs) are small-sized antibody fragments which can be selected against specific antigens starting from libraries of high diversity. ScFvs can be provided with signals for localization in specific intracellular compartments, and expressed as intracellular antibodies (intrabodies) [1-4]. The mechanism of action of an intrabody is generally based on the specific binding to an intracellular antigen, which results in the alteration of protein-protein interactions and sometimes delocalization of the target antigen from the usual cell compartment, with subsequent prevention of its activity [5].

It is now well accepted that intrabodies with proven effectiveness in inhibiting the function of specific endogenous targets, represent powerful tools for research studies and therapeutic applications [6, 7].

We explored an intrabody-based approach to address the issue of therapy for HPV-associated pre-cancerous and cancer lesions. The association of the HR genotypes of HPVs with several kinds of human cancer has been universally recognized since many years [8]; cervical cancer (CC) is the most severe for incidence and mortality rate [9], and genotype 16 (HPV16) is the most frequent, with almost 60% of CC cases worldwide [10].

The two currently available HPV vaccines, both including the HR 16 and 18 genotypes, are highly effective in preventing the HPV infection but, unfortunately, a decreased cancer incidence is not expected before few decades at least because of the long viral persistence successive to the infection and in view of the difficulty to vaccinate all the women [11]. For this reason, research studies worldwide are focusing on therapeutic interventions for the early lesions, in order to prevent tumor progression and invasiveness and avoid costly follow up. Several approaches designed to activate the immune response towards HPV cancers can be considered as therapeutic vaccines [12]. Other therapeutic strategies are aimed at hampering expression or function of the E6 and E7 viral proteins in view of their being responsible for the oncogenic activity of the HR HPVs [13-15].

E6 and E7 exert different and concerted pro-tumor actions mainly based on their capacity to target cellular proteins involved in the control of cell homeostasis through proteasome-mediated degradation, prevention of their interaction with other cellular proteins or alteration of their intracellular localization [16]. The E7 protein binds to pRB and displaces the E2F transcription factor, thus facilitating S-phase entry [17, 18]. E7 also associates with p21, HDACs and cyclins, resulting in alteration of their function [19].

The number of E6 cellular targets is increasing day by day together with the cell pathways potentially affected [20]. On a molecular basis, the E6 intracellular targets can be divided in two groups according to the presence of specific aminoacid sequences: LxxLL motifs or PDZ domains, respectively. The interaction of E6 with the E6AP ubiquitin ligase and p53 tumor suppressor in a trimeric complex belongs to the first group; such association causes p53 degradation and increases E6 stability [21-25], whereas its inhibition influences p53 activity and can cause p53 rescue [26-28].

The PDZ-domain binding site is located at Carboxy-terminus of the E6s belonging to HR HPV genotypes. The interaction with PDZ-containing proteins mainly involves the control of cell polarity and cell-cell adhesion, and the regulation of diverse cell signaling pathways; inhibition of this interaction has consequences relevant in the late stages of malignant progression [29-32]. Recently, the possibility of targeting both LxxL and PDZ oncogenic functional sites at the same time was investigated with therapeutic purpose [33].

Overall, the concerted activities of E6 and E7 influence cell cycle control, cell growth regulation and resistance to apoptosis, with the consequence of immortalizing and transforming human keratinocytes efficiently.

In previous studies from our group, three scFvs against the HPV16 E7 (16E7) were selected from phage-displayed antibody libraries [34] and expressed as intrabodies in different cell compartments. Evidence for their antiproliferative activity was obtained in HPV16-positive-infected cell culture systems [35-37]. Recently, one of these intrabodies (43M2SD), retained in the endoplasmic reticulum (ER), was shown to block the growth of HPV tumors in mouse models [38].

In the wake of these studies, we planned to investigate the effect of intrabody-mediated interference with the E6 activities on survival of HPV16-positive cells *in vitro* as well as on development of HPV tumors in preclinical models.

We selected an intrabody (I7) against the 16E6 by IACT, which allows the efficient and direct selection *in vivo* of stable intracellular binders for a specific antigen [39-43]. The I7 intrabody was provided with the signal for localization in cell nucleus (NLS) and expressed in cell cultures as I7nuc.

Herein, we demonstrated by confocal microscopy that I7nuc always co-localizes with E6, and is even able to modify the intracellular distribution of the oncoprotein. The intrabody-mediated perturbation of E6 interactions with cellular targets results in a significant decrease of cell survival mainly due to a necrotic process. Importantly, we showed that I7nuc intrabody holds antitumor activity, at least in two preclinical models for HPV-associated tumors.

## RESULTS

### IACT selection of I7 and expression and intracellular distribution of the I7nuc intrabody

The intracellular antibody scFv I7, specific for the 16E6 protein, was selected by IACT from a single pot library of intracellular antibodies (SPLINT), that is a murine naïve library of scFv fragments expressed in the yeast cytoplasm [42]. Selection was performed as described in Material and Methods section. According to specificity and antibody sequence integrity determined by DNA sequencing, scFv I7 was chosen for further analysis

**Figure 1:**
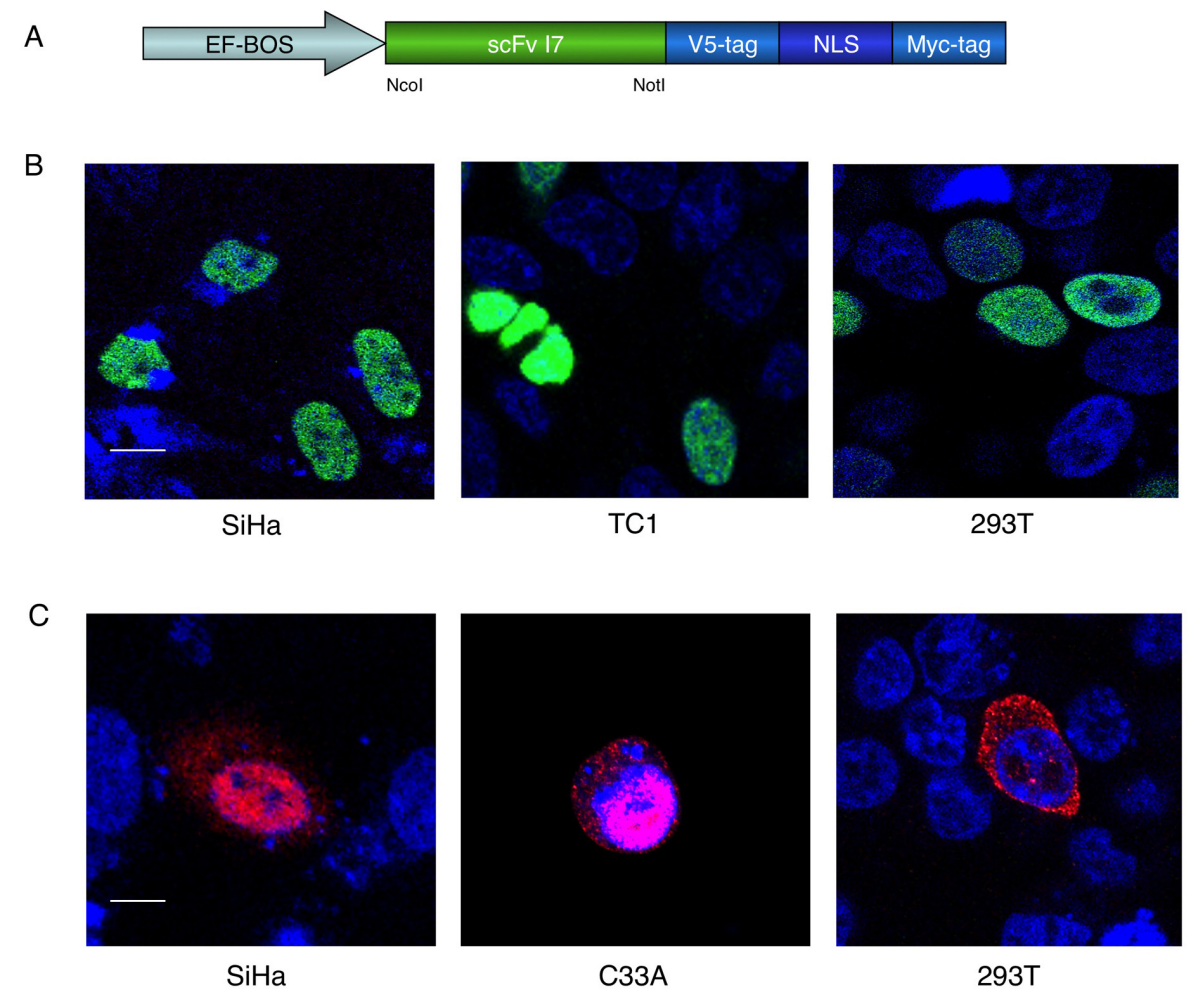
Intracellular localization of the I7nuc intrabody and 16E6 protein in HPV16-positive and HPV-negative cells **A.** Schematic representation of ScFvExI7nuc plasmid. The I7 coding sequence under control of the EF-BOS promoter, the V5-tag and Myc-tag for immunological detection, and the Nuclear Localization Signal (NLS) are shown. **B.** Confocal imaging of I7nuc expression. HPV16-positive SiHa and TC1 cells or HPV-negative 293T cells were transfected with ScFvExI7nuc plasmid. At 48 h post transfection, I7nuc expression was visualized by immunofluorescence microscopy using anti-V5 mAb (green). Nuclei are displayed in blue. The merge image shows overlay of the two fluorochromes. **C.** Confocal imaging of exogenous 16E6 expression. Cervical cancer SiHa and C33A cells or 293T cells were transfected with HAE6 pcDNA3 plasmid. The expression of 16E6 was visualized at 24 h post-transfection using anti-HA mAb (red). Nuclei are displayed in blue. Magenta stain in the merge images indicates the nuclear localization of 16E6. The white bar represents 10 μm of micron scale bar.

Since E6 is a modulator of transcriptional activity and because many of its targets related to transforming ability are located in the cell nucleus of HPV16-positive cells, the I7 intrabody was provided with the signal for nuclear localization (NLS). To do this, the I7-coding sequences were cloned in the ScFvE-nuclear eukaryotic nuclear vector of the ScFvExpress series [3], obtaining the ScFvExI7nuc plasmid (schematically represented in Figure 1, panel A).

To verify expression and integrity of the intrabody molecules, human embryonic kidney 293T cells were transfected with the ScFvExI7nuc plasmid. WB of cell lysates with anti-V5 mAb revealed the presence of an I7nuc protein with an estimated MW of about 30 KDa, as expected for scFv molecules inclusive of NLS (data not shown).

To confirm the nuclear localization of I7, HPV16-positive SiHa and TC-1 cells as well as HPV-negative 293T cells and C33A keratinocytes, were transiently transfected with the ScFvExI7nuc plasmid. At 24 or 48 hours post-transfection, cells were fixed and incubated with anti-V5 mAb. Immunofluorescence and confocal microscopy analysis showed a diffused intranuclear accumulation of I7nuc in all the cell lines, and I7nuc expression levels from low to high could be observed thanks to remarkable stability of the intrabody (Figure 1, panel B).

### Effect of I7nuc expression on intracellular E6 localization

In agreement with the multiple localizations of its interactome, the 16E6 has been shown to localize in both cell nucleus and cytoplasm [44-47]. Thus, we were interested in investigating the effect of the I7nuc expression on the intracellular E6 distribution to gain further insight into functional relationships that might correlate with the E6 localization. Owing to its rapid turnover in HPV16-positive cells [48], E6 is hardly detectable by immunofluorescence and confocal microscopy analysis without treatment with proteasome inhibitors. Therefore, SiHa cells were transiently transfected with E6HA, a 16E6-expressing pCDNA plasmid, alone or in combination with the ScFvExI7nuc plasmid. The HPV-negative C33A and the 293T cells were transfected in parallel to highlight possible differences ascribable to HPV genome expression or cellular context. The E6 expression was analyzed at 24 h and 48 h post-transfection. As shown in Figure 1C, overexpressed HAE6 was distributed in both cell compartments, with a predominant nuclear localization in SiHa and C33A cells. Consecutive optical sections showed that the level of E6 expression seems to influence its intracellular distribution in transfected epithelial cells, because at high expression levels the HAE6 accumulated also within the cytoplasm. Conversely, in 293T cells, the HAE6 protein was predominantly distributed in the cytoplasm and faintly visible in patches in few nuclei, suggesting that E6 nuclear import could be cell type-dependent.

**Figure 2:**
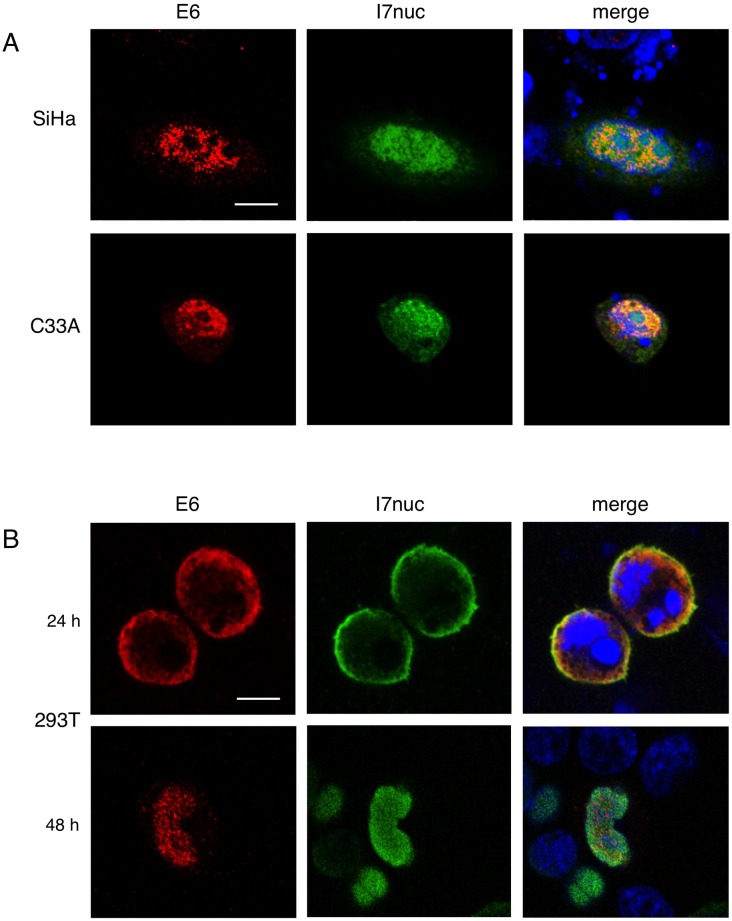
Colocalization of 16E6 and I7nuc in HPV16-positive and HPV-negative cells **A.** Representative confocal imaging of SiHa and C33A cells 48 h after co-transfection with plasmids expressing HAE6 (red) and I7nuc (green), revealed with anti-HA and anti-V5 mAbs, respectively. **B.** 293T cells were stained 24 (upper panel) and 48 (lower panel) hours after transfection with HAE6 (red) and I7nuc (green)-expressing plasmids. Nuclei are displayed in blue. Overlay of the three fluorochromes is shown (merge). White bars represent 10 μm of micron scale bar.

Immunofluorescence and confocal microscopy analysis performed after double transfection, highlighted the colocalization of E6 and I7nuc in the same cell compartment, regardless of whether the expression was nuclear or cytoplasmic, in all the cell lines analyzed (Figure 2A). This finding suggests that I7nuc could recognize both nuclear and cytoplasmic E6 forms.

Interestingly, at 24 h post-transfection, E6/I7nuc complexes are localized exclusively in the cytoplasm of 293T cells, but they accumulate mostly in cell nucleus at longer times, suggesting that the NLS sequences of the intrabody could promote the E6 relocalization (Figure 2B).

### Effect of I7nuc on p53 levels

As above described, promotion of p53 ubiquitination and subsequent degradation are the best recognized activity of 16E6 [23].

**Figure 3:**
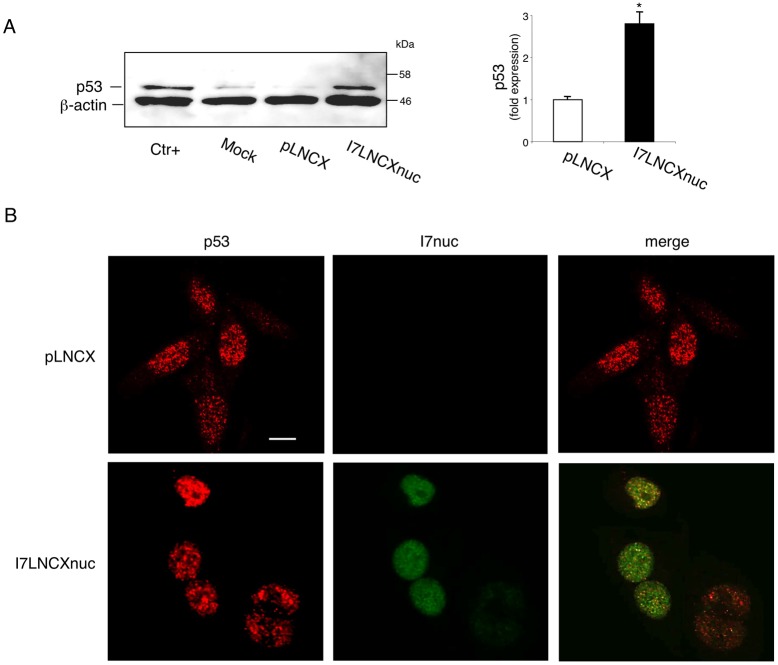
I7nuc expression inhibits the ability of 16E6 to mediate p53 degradation **A.** HPV16-positive SiHa cells were infected with I7LNCXnuc or empty pLNCX retroviral vectors. After 24 h, whole cell lysates were analyzed by Western blotting using anti-p53 antibody. C33A cell lysate was used as a positive control (Ctr+). The levels of β-actin are shown as loading controls (left panel). Relative quantities of p53 were normalized to β-actin protein and presented as fold expression with respect to the levels in non-transduced cells, which was arbitrarily set to 1 (right panel). The bars represent the mean ± standard deviation (SD) of three independent experiments. *, *p* < 0.05. **B.** Intracellular p53 localization (red) was determined by confocal microscopy analysis in I7nuc-expressing (green) and non-expressing SiHa cells. The merge image shows colocalization of p53 with the I7nuc intrabody. The white bar represents 10 μm of micron scale bar.

To elucidate whether the I7nuc binding to E6 could alter functionality of the oncoprotein, we assayed the ability of I7nuc to inhibit the E6 capacity to promote p53 degradation in SiHa cells. The retroviral expression system based on pLNCX vectors was employed to obtain intrabody expression in the highest number of cells. At different times after infection, cells were lysed and the level of p53 in each sample was assessed using an anti-p53 antibody in WB assay. As shown in Figure 3 (panel A), an increase in p53 levels was observed at 24 h post-infection, indicating that the I7 binding can partially reduce the 16E6 ability of degrading p53.

The intracellular distribution of endogenous p53 was then investigated by confocal microscopy in HPV16-positive SiHa cells infected with the intrabody-expressing retroviruses. Although p53 was mainly located in cell nucleus of both I7nuc-expressing and non-expressing cells, a weak punctuate staining was observed in the cytoplasm of the control cells. Interestingly, we observed a re-distribution of p53 from cytoplasm to nucleus in the I7nuc-expressing SiHa cells (Figure 3, panel B).

### I7nuc expression decreases the growth of HPV16-positive cells *in vitro*

**Figure 4:**
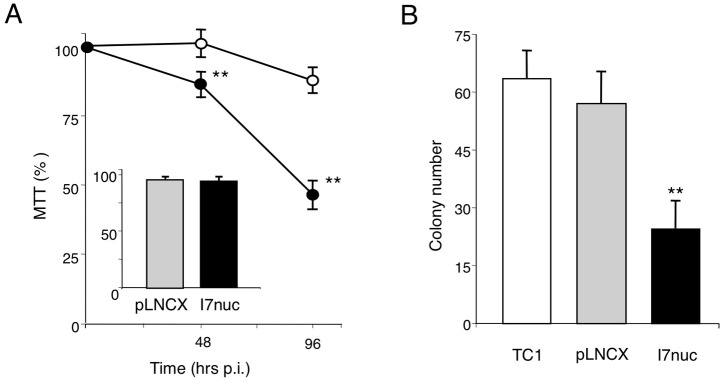
Effect of I7nuc expression on cell proliferation **A.** HPV16-positive SiHa cells and HPV-negative C33A cells were mock-infected or infected with pLNCX or I7LNCXnuc retroviruses. Cell viability was evaluated by MTS assay at 48 and 96 h post-infection for I7nuc-transduced (filled circles) and pLNCX-transduced (open circles) SiHa cells, and at 96 h post-infection for C33A cells (inset). Data are expressed as a percentage of MTS conversion in non-transduced cells and represent the mean +/− SD of samples in triplicate from one representative experiment of three with similar results. **, *p* < 0.01. **B.** Clonogenic assay of TC-1 cells mock-infected or infected with pLNCX or I7LNCXnuc retroviruses. Data represent the mean ± SD of samples in triplicate from one representative experiment of two with similar results. **, *p* < 0.01.

To determine whether the retroviral vector-mediated I7nuc expression impacted on proliferation of cancer cells, we examined the growth of HPV16-positive or HPV18-positive and HPV-negative cells at different times after transduction by MTS assay. As shown in Figure 4 (panel A), we found that the I7nuc expression significantly reduced proliferation of SiHa cells with respect to the pLNCX virus-infected control cells, and this reduction was more evident at late times after transduction. In fact, if we consider proliferation of the non-infected cells as 100%, inhibition ranged from 19% to 50% in the interval from 24 to 96 h post-infection. Similar results were obtained for the infected TC-1 and C3 tumor cells (data not shown). Conversely, no inhibition of cell growth was observed when the HPV-negative C33A cells (Figure 4, panel A, insert) or HPV18-infected HeLa cells (Figure S1) were infected with the same retroviral vectors, indicating that inhibition of cell growth was specific for the HPV16-positive cells. Specificity of the I7nuc antiproliferative activity was also demonstrated by the absence of growth inhibition in SiHa cells transfected with non-relevant R4nuc (OD_490_= 0.82± 0.11) as compared to those transfected with scFvExI7nuc plasmid (OD_490_= 0.56± 0.18). To confirm the effect of I7nuc expression on cell growth and possibly discriminate between cell growth inhibition and cell killing, a clonogenic assay was further performed on HPV16-positive cells after mock or retrovirus infection. The results showed that I7nuc expression significantly lowers the clonogenic potential in all the tumor cells analyzed. In Figure 4, panel B, the results for I7nuc-expressing TC-1 cells in comparison with TC-1 cells non infected or infected with the pLNCX virus, are shown.

These findings demonstrated that I7nuc expression significantly inhibits the growth of HPV-16 positive cells *in vitro*.

### I7nuc expression induces late apoptotic and necrotic death of HPV16-positive cells *in vitro*

**Figure 5:**
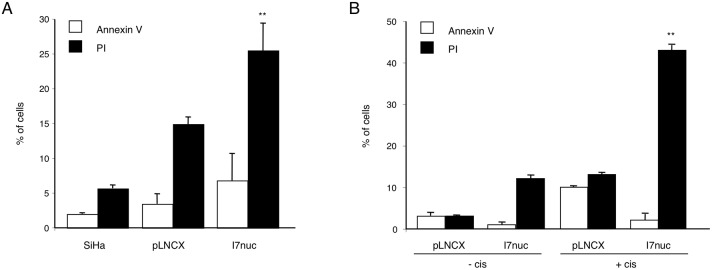
I7nuc causes cell death of HPV16-positive cells **A.** HPV16-positive SiHa cells were infected with pLNCX or I7LNCXnuc retroviruses. At 48 h post-infection the percentage of early apoptotic (□ AnnexinV+/PI-) and late apoptotic/necrotic cells (■ AnnexinV+/PI+, AnnexinV-/PI+) was determined by cytometry after AnnexinV-FITC/PI staining. Data represent the mean ± SD of four independent experiments. **, *p* < 0.01. **B.** I7LNCXnuc or pLNCX-infected SiHa cells were treated with 5 μM cisplatin. After 24 h, cell death was analyzed by measuring the percentage of early apoptotic and late apoptotic/necrotic cells. Data represent the mean ± SD of three independent experiments. **, *p* < 0.01.

In order to investigate whether the growth-inhibitory effect of I7nuc expression on HPV16-positive cells was associated with cell death, the percentage of apoptotic/necrotic cells was measured by cytometry analysis after AnnexinV-FITC/PI staining of SiHa cells infected with I7LNCXnuc or pLNCX retroviruses. The results showed that I7nuc expression barely induced early apoptosis (AnnexinV+/PI-) whereas it was associated with a significant increase of late apoptotic/necrotic cells (AnnexinV+/PI+, AnnexinV-/PI+) in comparison to SiHa cells infected with empty pLNCX viral vector (Figure 5, panel A). The absence of undergoing apoptosis in I7nuc-expressing HPV16-positive cells was further confirmed by WB detection of uncleaved PARP-1, and by the absence of DNA fragmentation as revealed by ELISA (data not shown).

The effect of intrabodies on cell survival was further examined in pLNCX or I7nuc-infected SiHa cells treated with 0.1 μM cisplatin or control diluent, for 24 h. Apoptosis and necrosis were determined by AnnexinV-FITC/PI staining and cytofluorimetric analysis. As expected, treatment with cisplatin was effective in inducing both early and late apoptosis in the control pLNCX-infected SiHa cells; interestingly, a highly consistent increase in the percentage of late apoptosis/necrosis was observed in the I7nuc-expressing cells (Figure 5, panel B).

### I7nuc antitumor effect *in vivo*

In view of our previous findings, and in consideration of the tight interconnection between the E6 and E7 activities, we investigated the possibility that even the anti-16E6 intrabody could exert an antitumor activity *in vivo*. To this aim, we used the same preclinical HPV tumor models previously employed for evaluation of the antitumor activity of an anti-16E7 intrabody [38].

TC-1 and C3 cells were infected with pLNCX or I7LNCXnuc retroviruses. Before injection into animals, I7nuc expression was monitored by immunofluorescence microscopy 24-48 h post-infection. Intrabody expression was obtained in more than 40% of the cells, which corresponds to the average percentage of infection that we obtained in these cell systems. Uninfected, I7LNCXNuc- or pLNCX-infected cells were injected subcutaneously in C57BL/6 mice. Mice were monitored for tumor development and those showing a delay of tumor onset with respect to the controls were observed for a further 1 month after suppression of the controls for ethical reasons.

We could observe similar survival curves in both mouse models. In fact, the mice of the control groups injected with uninfected or pLNCX-infected TC-1 or C3 cells developed tumors 2-3 weeks post inoculum. All the TC-1 tumors from I7nuc-expressing cells (I7nuc-TC-1) grew with a significant delay with respect to the controls. In fact, 20% of mice receiving the I7nuc-TC-1 developed tumor at day 31, 40% at day 61 and the residual 60% remained tumor-free all over 9-weeks of the observation time. In contrast, 20% of control mice developed tumor at day 17, whereas 100% of them were ill at day 20 as well as the mice injected with pLNCX-infected cells. In the C3 model, the effect was less significant particularly in the long run, as 40% of the mice treated with the I7nuc-expressing cells (I7nuc-C3) developed tumor at day 20, and 60% at day 35. Control mice of this series all developed tumor at day 17 whereas 20% of mice injected with pLNCX-infected cells developed tumors at day 17 and all of them were ill at day 20.

**Figure 6:**
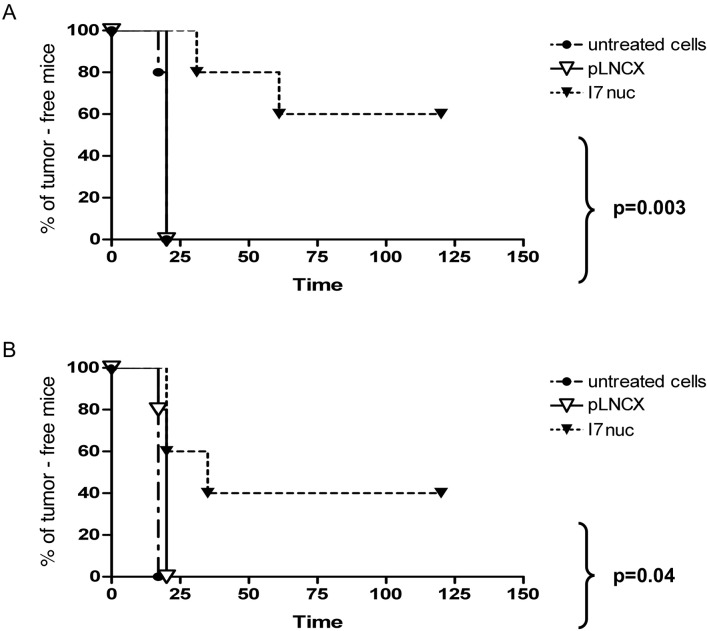
Inhibition of TC1 and C3 tumor growth by I7nuc intrabody expression Kaplan-Meier analysis of time-to-tumor development of C57BL/6 mice injected subcutaneously in the right leg with 5×10^4^ TC-1 (panel A) or with 5×10^5^ C3 (panel B) tumor cells. Both cell lines were uninfected, infected with control retroviral particles only (pLNCX) or with retroviral particles expressing the anti-E6 intrabody (I7nuc) prior to injection in mice. Log-Rank test among Kaplan-Meier curves indicate statistically significant differences (*p* < 0.005).

Log-Rank test among Kaplan-Meier curves indicated statistically significant differences for the I7nuc-TC-1- and I7nuc-C3-injected mice and pLNCX controls, with *p* = 0.003 and *p* = 0.04, respectively (Figure 6). Furthermore, we observed that tumors developed from the I7nuc-infected cells, were overall more fluid than those developed in the controls, making us to hypothesize that necrotic processes can take part in tumor thus treated (Venuti, personal communication).

## DISCUSSION

Antibody technologies have been proved capable of generating powerful therapeutic agents for a wide variety of diseases including cancer [4, 6, 7]. Increasing attention has been recently given to intrabodies, recombinant antibody fragments engineered to be expressed inside the cells, that can hold exquisite specificity combined with high affinity, stability and solubility. Intrabodies have significant potential for the therapy of human diseases in which etiologically relevant proteins or protein-protein interactions are found inside the target cells [4, 51].

The tight relationship between HPV oncogenic activity and fundamental intracellular protein-protein interactions, provides the opportunity to disrupt such interactions and counteract tumor progression, and makes HPV the ideal target for an intrabody-based therapy.

In particular, the key involvement of E6 in cervical cancer pathogenesis makes it a very attractive target for the development of intrabodies designed to suppress the E6 functions. Two scFv fragments recognizing the N-terminal region of 16E6 and able to inhibit the formation of the 16E6/p53 complex were previously described [52]. Successively, two intrabodies against 16E6 have been reported and shown to cause apoptosis following expression in cervical cancer cells [53, 54].

In the present study, we report the development and characterization of a novel intrabody in single-chain format (scFv) against the 16E6 oncoprotein. ScFvs that binds to the 16E6 protein were isolated using a SPLINT in the yeast IAC technology [41]. In this system, antigen-specific intrabodies can be isolated directly from gene sequences with no need of purifying the corresponding proteins, and only following antigen recognition in the intracellular environment. Also, this technology ensures thermodynamic stability, which represents a fundamental characteristic for the proper functioning of a scFv, particularly as an intrabody, as a long life-span implies increased chances to meet its antigen and carry out its function in both therapeutic and diagnostic applications. In the reducing environment of cell cytoplasm, the higher intrinsic stability of a IACT-selected intrabody can balance the intrabody loss of disulphide bonds [42, 51].

Since many of the E6 functions are performed in cell nucleus, the scFv I7 intrabody was provided with a signal for nuclear localization (I7nuc). The correct intracellular localization of I7nuc was verified by immunofluorescence and confocal microscopy analysis in HPV16-positive cells such as the immortalized SiHa and the tumor TC-1 cells, and in the HPV-negative 293T and C33A cells (Figure 1B). The proper nuclear compartmentalization indicates that the selected antibody has the appropriate solubility to be transported from the cytoplasm to the nucleus, expected in the light of the selection methodology adopted.

As a part of this study, we examined the intracellular localization of E6 alone or in combination with I7nuc, both expressed from transfected plasmids in HPV16-positive and HPV-negative cells. In agreement with previous studies [44-47, 55], we found that in SiHa and C33A cells, the exogenous 16E6 was mainly nuclear, even though some amount of E6 was present also in cytoplasm, suggesting that the oncoprotein may shuttle between the two compartments. Although the positive red fluorescence in the cytoplasm may support the E6 binding to its numerous cytoplasmic targets, the possibility that it is ascribable to oligomeric forms of E6 cannot be excluded. In fact, previous studies showed that full-length E6 is prone to self-association *in vitro* and, in spite of three NLSs, can remain in the cytoplasm as large homo- or hetero-oligomeric forms when its concentration exceeds the capacity of nuclear translocation and target interactions [56, 57]. Accordingly, in 293T cells, where higher expression levels can be easily achieved, the E6 was almost exclusively distributed in cell cytoplasm, and only faintly in some cell nuclei.

In the double-transfected cells, the common denominator appeared to be the colocalization of E6 and I7nuc in both subcellular compartments, either nucleus or cytoplasm, that is meaningful of an interaction between the two proteins. Notably, in SiHa and C33A cells, a fraction of I7nuc remained in the cytoplasmic compartment, indicating that the I7 nuclear localization might be prevented by the formation of E6-I7nuc cytoplasmic complexes. However, such E6-I7nuc interaction occurring in the cytoplasm seems to be specific for the epithelial cells. In fact, in 293T cells, E6-I7nuc complexes are present essentially in the cytoplasmic compartment, where E6 is found when expressed alone, while they move to the nucleus at longer times post-transfection. This observation may indicate a role of cargo for I7nuc, that would be able to relocate E6 to the nuclear compartment, with possible consequences on the cytoplasmic E6 functions in the natural systems.

Structural studies using surface plasmon resonance, circular dichroism and fluorescence spectroscopies supported with important details the current knowledge about the E6 mechanism of action. A model was proposed in which the N-terminus recruits a ubiquitin ligase to mediate protein degradation, while the C-terminus would be important for the binding of p53 and other target proteins such as hDlg PDZ [58]. Furthermore, it was demonstrated *in vitro* but not yet *in vivo*, that E6 dimerizes through its N-terminal domain and such dimerization promotes the p53 polyubiquitination by E6AP even though it is not sufficient for p53 degradation [59]. Then, determination of crystal structure of 16E6 revealed that two zinc domains and one linker helix form a basic-hydrophobic pocket which captures helical LxxLL motifs and is responsible for the E6 oncogenic activities. Importantly, the E6 N terminal self-association interface, the LxxLL binding region and the PDZ domain- binding motif at the extreme C terminus of E6, have been mapped onto the structure of 16E6 and showed not to overlap to each other [60].

As we do not know the actual I7nuc binding site on E6, we cannot exclude an overlapping with the N-terminal region responsible for the E6 dimerization. The I7nuc binding to this region might hamper the E6 dimerization and contribute to circumvent the p53 degradation while retargeting E6 to the nucleus as observed in the SiHa cells system.

In the light of the anti-proliferative and pro-apototic activity of p53, I7nuc was expressed in cervical cancer cells by retroviral system to study the possible effect on p53 degradation as well as on proliferation and survival of HPV16-positive cells. I7nuc was able to induce an increase of p53 levels as previously reported for other anti-E6 intrabodies [52, 53]. However, in our experimental conditions, we could not observe a sharp inhibition of p53 degradation, suggesting that the I7nuc-binding site could only partially overlap with the LxxLL binding region on E6. In alternative, it is possible that I7nuc does not work by preventing directly the binding E6AP-E6, but rather restraining E6 into the nucleus thus hampering formation of E6 dimers and export of p53 from nucleus to cytoplasm and consequent degradation. The confocal microscopy observation is in agreement with this interpretation, as the I7nuc expression seems to interfere with the cytoplasmic localization of p53. However, we cannot exclude that the modest p53 rescue may simply result from an increased stability due to the I7nuc interference with the interaction between E6 and p300 acetyltransferase or CREB-binding protein [61, 62].

In agreement with Griffin et al [53], we found that the anti-E6 intrabody expression adversely affected the replicative potential of HPV16-positive cells as revealed by progressive inhibition of their proliferation and survival. The I7nuc activity was specific since the growth of infected C33A and HPV18-positive HeLa cells was unaffected.

Importantly, we found that the reduced replicative potential of the I7nuc-expressing cells depended on enhanced cell death. However, in contradiction with the results previously obtained by other groups [53, 54], apoptosis was slightly induced in I7nuc-expressing SiHa cells, and a relevant amount of necrotic cells was observed at 48 hrs post-infection. Necrosis was also the cell death pathway essentially observed in I7nuc-expressing SiHa cells in the presence of DNA damage (Figure 5).

Although necrosis was considered as an accidental and uncontrolled type of cell death, several recent evidences indicate that its course might be tightly regulated by programmed pathways such as mitochondrial dysfunction, enhanced generation of reactive oxygen species, ATP depletion, proteolysis by calpains and cathepsins, and early plasma membrane rupture. In addition, necrosis can represent a default cell death pathway when proteins involved in apoptosis are inhibited [63]. In this context, E6 binding to Fas-Associated protein with Death Domain (FADD) and pro-caspase 8 [64] as well as E6-mediated degradation of pro-apoptotic Bax protein [65] could block apotosis and induce the observed necrosis in HPV16-infected cells.

The antitumor efficacy of I7nuc was also investigated *in vivo* using the TC-1 and C3 mouse models described elsewhere [38, 66]. In the present as well as in the previous study concerning an anti-E7 intrabody, such models were employed to assess *in vivo* the possible antitumor activity of a binder against the oncogenic activity of a protein, rather than to study the host immunological response. Therefore, the intrabody was delivered to the mouse tumor cells by retroviral system prior to subcutaneous cell injection into animals. The best time of mice injection was identified in the range between 24 and 48 hours after infection, when the maximum of intrabody expression is reached and the infected cells are proliferating and still countable. The mice were then monitored for tumor onset and development.

The results indicated that, in both tumor models, the expression of I7nuc could either delay or hamper tumor development, supporting the antitumor potential of the intrabody. The *in vivo* experiment showed that the effect of the anti E6 intrabody on C3 tumor growth (Figure 6, panel A) was lower than that on TC-1 cells (Figure 6, panel B). This difference probably depends on the rate of E6 synthesis, driven in C3 cells by a natural promoter and in TC-1 cells by a viral promoter.

Overall, our results suggest that the reduction in tumor size and development observed in mice could be ascribable mainly to inhibition of cell growth and survival due to the infection with I7nuc retrovirus.

In conclusion, we showed that nuclear expression of an E6-targeted intrabody can hamper effectively and specifically the E6 oncogenic functions. This is the first demonstration *in vivo* of the antitumor activity of an anti-16E6 intrabody and is particularly intriguing in consideration of the antitumor activity previously obtained in our laboratory using an anti-16E7 intrabody [38]. Co-administration of anti-E6 and anti-E7 intrabodies could offer, for the first time, the possibility to counteract the oncogenic activities of both E6 and E7 and design a rational molecular therapy for the HPV cancer. We believe that, once solved the problem of a safe delivery to humans, our studies could lead to the development of safe and specific drugs addressing the need of a non-invasive treatment particularly for HPV-associated early lesions.

## MATERIALS AND METHODS

### IAC technology for antibody selection

Construction of the SPLINT library was previously described [42]. Library diversity was estimated to 10^7^ by counting the obtained number of clones with <0.01% of clones with no-insert ligation. The library was prepared from the glycerol stock of *E. coli* DH5αF' containing the scFv gene library. An aliquot corresponding to 50 times the diversity of the library was used to prepare the recombinant plasmid DNA using the QIAGEN Plasmid Maxi Kit (Qiagen, Hilden, Germany) that was subsequently used for the transfection of L40 yeast cells.

IACT identifies physically interacting antibody-antigen pairs exploiting the interaction between target proteins fused in-frame to the DNA-binding domain of the *E. coli* lexA protein and the scFv library fused to the activation domain of the herpes simplex virus VP16 protein, co-expressed in the *S. cerevisiae* L40 reporter strain. For IACT selection, HPV16 E6 sequence was PCR amplified by the EcoRIdir/E6 SalIrev couple of primers from the previously obtained E6/pGEMT plasmid carrying the whole E6 sequence (unpublished), and cloned in pMICBD1 digested with EcoRI/SalI.

The primer sequences with the restriction sites underlined, are as follows:

EcoRIdir: 5′ GGCCGAATTCATGCACCAAA AGAGAACTGC 3′

E6SalIrev: 5′ GTCGACCCCGGGTTACAGCT GGGTTTCTCTACGTG 3′

Before selection of SPLINT library, 16E6 bait was tested for transactivation of reporter genes as previously described [42]. The 16E6 bait resulted to be self-activating.

Determination of the minimal inhibitory concentration of the competitive inhibitor of HIS3 3-amino triazole (3-AT), which is needed for the bait to suppress self-activation growth whereas maintaining detectable true interactions, was performed. Ten mM 3-AT was found to be the optimal concentration to suppress the background and was therefore used in the interaction screening. The SPLINT library was transformed in the yeast L40 containing 16E6 expressing bait by using a rearranged lithium acetate transformation protocol [43]. Trasformants were selected for histidine prototropy and screened for LacZ activity. Plasmid DNA was isolated from 22 strong blue colonies and analyzed by BstNI-fingerprinting, yielding 15 different scFv fragments. These were individually tested in secondary yeast screening, yielding two positive clones specifically interacting with 16E6 bait and not interacting with lamin bait used as an irrelevant antigen.

### Design and construction of the I7nuc plasmids

In order to provide scFv I7 with the signal for nuclear localization (NLS), the I7 coding sequence was PCR amplified using the anti-E6 dir and anti-E6 revHind couple of primers, and cloned in the pGEMT vector, obtaining the I7 pGEMT plasmid. The primer sequences with the BamHI and HindIII restriction sites underlined, are as follows:

anti-E6 dir: 5′ GCGCGGATCCGATATTGTGA TGACCCAGTC 3′

anti-E6 revHind: 5′ GCAAGCTTGCGGCCGCAG TACTATCCAGGCCCAG 3′

I7 pGEMT was then double digested and the NcoI/NotI fragment obtained was cloned in the scFvEx-nuclear vector digested with the same enzymes, obtaining ScFvExI7nuc. The I7 sequence including the NLS was then PCR amplified (95°C for 1 min, 50°C for 1 min, 74°C for 1 min, 35 cycles) from scFvExI7nuc using the aE6LNCX dir and ClaNuc rev couple of primers, and cloned in the pGEMT vector, obtaining I7nuc pGEMT. The primer sequences, with the HindIII and ClaI restriction sites underlined, are as follows:

aE6LNCX dir: 5′ GCGCAAGCTTCCATGGCCA GCGCGCATGCC 3′

ClaNuc rev: 5′ CCCGGATCGATCTATGCG GCCCCATTCAGATCC 3′

The I7nuc pGEMT was then used to transform the DH5alfa (*Dam-),* and the HindIII/ClaI fragment was eluted and cloned in the pLNCX retroviral vector digested with the same restriction enzymes, obtaining I7LNCXnuc.

All clones were checked by sequence analysis.

### Cell lines and cell transfection

The human cervical carcinoma cell lines SiHa (ATCC HTB-35) and C33A (ATCC HTB-31) were grown at 37°C in humidified atmosphere with 5% CO_2_ in Dulbecco's modified Eagle's medium (DMEM, Gibco, UK) supplemented with 10% heat-inactivated fetal calf serum (FCS), 100 units/ml penicillin, 100 mg/ml streptomycin and 2mM glutamine. SiHa cells are keratinocytes harboring the HPV16 genome and expressing the E6 and E7 proteins, whereas C33A cells are keratinocytes negative for HPV.

The human embryonic kidney 293T (ATCC CRL-1573) and Phoenix cells (ATCC 3444), used for transfection and production of retroviral particles respectively, were cultured under standard conditions. Cell lines were subcultured for a maximum of 12 passages starting from the original stocks authenticated by Istituto Nazionale per la Ricerca sul Cancro (Genova).

The murine TC-1, derived from primary lung epithelial cells co-transformed by HPV16 E6/E7 oncoproteins and c-H-Ras [49] and C3 cell line, derived from embryonic mouse cells transformed with the full HPV16 genome and activated ras oncogene [50], were grown in RPMI 1640 with 10 mmol/L of HEPES, 1 mmol/L of sodium pyruvate supplemented with 2 mmol/L of nonessential amino acids and 10% FCS. Both tumor cell lines are passages of the original clones and were routinely checked for the presence of HPV sequence and resistance to the G418 antibiotic selection (0.4 μg/ml). Both are able to establish subcutaneous tumors in C57BL/6 syngenic mice, which are used as models of human HPV16-associated neoplasms.

One day before transfection, cells were trypsinized and seeded into six-well plates. Cells were then transfected with the HAE6 and ScFvExI7nuc plasmids, alone and in combination, or with R4nuc [35], expressing the irrelevant anti-β-galactosidase intrabody, by jetPEI^®^ transfection reagent (Polyplus transfection, Illkirch, France) according to the manufacturer's instructions.

### Production of I7nuc-expressing retroviruses and cell infection

Retroviral particles production was performed as previously described [38]. Briefly, Phoenix cells, containing the stably integrated gag, pol and env genes of the Moloney leukaemia virus necessary for retrovirus assembly, were transfected with the recombinant I7LNCXnuc or the empty pLNCX viral vector as a control. Forty-eight hours after transfection of Phoenix cells, the virus-containing cell culture supernatant was concentrated 100X by RetroX concentrator (Takara Clontech, CA, USA) and used to infect cells at 60% of confluence.

### Immunofluorescence and confocal microscopy

Cells were fixed with 3.7% paraformaldehyde in PBS for 20 min at room temperature, permeabilized with 0.1% Triton X-100 in PBS for 5 min and then incubated for 1 h with mouse anti-V5 tag mAb (Life Technologies) followed by decoration with goat anti-mouse IgG fluorescein-conjugated (ICN Cappel Inc), and with rabbit anti-HA policlonal antibody (Sigma-Aldrich) followed by decoration with goat anti-rabbit Alexa-fluor 594 (Life-technologies). RedDot^™^2 far-red nuclear stain (Biotium, Inc., Hayward) diluted 1:400 was utilized as a nuclear marker. For p53 protein detection, rabbit anti-p53 polyclonal Ab (Santa Cruz Biotechnology) followed by goat anti-rabbit Alexa-fluor 594 (Life-technologies) was used. Control incubations demonstrated no cross-reactivity between the anti-immunoglobulin conjugates or between the anti-immunoglobulin conjugate and irrelevant primary antibodies. All samples were examined using a confocal microscope Leica TCS SP5 and processed with LAS AF version 1.6.3 software (Leica Microsystems). To prevent cross emission spectra, specific lasers (488 nm, 546 nm and 633 nm) were activated in sequential mode to acquire the images.

### Western blot analysis

I7nuc- and pLNCX- infected SiHa cells were lysed with cold high salt extraction buffer, (50 mM Tris-HCl pH 7.5, 400 mM NaCl, 1 mM EDTA, 1 mM EGTA, 1% Triton, 0.5% NP-40 and 10% glycerol supplemented with 20 mM beta-glycerophosphate, 1 mM PNPP, 1 mM Na_3_VO_4_, 1 mM PMSF, 2 mM dithiothreitol and PIC; Sigma-Aldrich). Whole cell extracts (20 μg) were separated by 12% SDS-PAGE, and blotted to PVDF membrane (Immobilon P, Millipore, Billerica MA, USA). Unspecific binding sites were blocked by incubating the membranes for 1 h in 0.05% Tween 20 (v/v in TBS) supplemented with 5% nonfat milk, followed by overnight incubation at 4°C with primary antibodies specific for anti-p53 (DO-1, Novocastra, UK), anti-β-actin (Santa Cruz biotechnology) or anti-V5 tag mAb (Life Technologies), and decoration by peroxidase-labeled anti-mouse IgG (Sigma-Aldrich), or anti-goat IgG (Thermo scientific, Rockford IL, USA), (Sigma-Aldrich), and chemiluminescence detection (Luminata Crescendo Western HRP substrate, Millipore, Billerica MA, USA). Quantitative evaluation of proteins was determined by BioSpectrum Imaging system analysis using the VisioWorksLs software program (UVP, CA, USA).

### Cell viability and apoptosis analysis

Cell viability was investigated by MTS assay (CellTiter 96® AQueous One Solution Cell Proliferation Assay, Promega, Madison, USA) at 48 and 96 h post-infection with I7nuc- and pLNCX-expressing retrovirus in exponentially growing cell cultures. Cell viability was determined as the ratio between the absorbance obtained in test wells and the absorbance obtained in uninfected cells. Alternatively, I7nuc- and pLNCX-infected cells were analyzed by colony forming assay (CFA) performed 24 post-transduction. Cells were trypsinized, diluted 10, 100 or 1000 times, and grown for 8-15 days under G418 selection. To visualize the colonies, cells were washed in PBS, fixed and stained on the plates with crystal violet in 20% methanol.

For analysis of apoptosis, cells were infected as above and analyzed after AnnexinV-FITC/PI staining (Molecular Probes, Invitrogen) using the Tali™ Image-Based Cytometer (Life Technologies, Invitrogen. Apoptosis was also investigated using Cell Death Detection ELISA kit (Roche Diagnostics), according to the manufacturer's instructions.

### Assessment of antitumor effects in mouse models

Six-week-old female C57BL/6 mice were obtained from Charles River and maintained under specific pathogen-free conditions at the Experimental Animal Department of the Regina Elena National Cancer Institute (Rome, Italy). Animal experiments described in this study were performed according to the Institutional animal use guidelines and the Italian law DL 116/92. Before injection into mice, TC-1 or C3 cells were trypsin/EDTA treated, washed, re-suspended in saline solution, and adjusted to the right density for injection. Three groups of C57BL/6 mice were injected subcutaneously into the right inner flank with 5× 10^4^ I7nuc-TC-1 or 5× 10^5^ I7nuc-C3 cells. Uninfected or infected with retrovirus only (pLNCX) tumor cells were used as negative controls. Mice were observed daily until tumor onset and, after that, tumor burden was evaluated by palpation twice a week for about 3 months. In consideration of the ethical guidelines suggesting to minimize the number of experimental animals, 5 mice per treatment were used.

### Statistical analysis

Data were expressed as the mean ± standard deviation (SD). Statistical analysis was performed using the Student's test for unpaired data and p values of <0.05 were considered significant. For analysis of antitumor effect, data were analyzed by Log-Rank test among Kaplan-Meier curves and considered significant when *p* < 0.05.

## SUPPLEMENTARY MATERIAL FIGURE



## Abbrevaitons

CC: cervical cancer
CREB-binding protein: cAMP-response element-binding protein
DFF40: 40-kDa subunit of DNA fragmentation factor
E6AP: E6-associated protein
ER: endoplasmic reticulum
FHL2: Four and a Half LIM Domains 2
HDAC: Histone deacetylase
HPV: Human papillomavirus
HR: high risk
IACT: Intracellular Antibody Capture Technology
IRF3: Interferon regulatory factor 3
NLS: nuclear localization signal
OD: optical density
PDZ: post synaptic density protein
PIC: Protease Inhibitor Cocktail
PMSF: phenylmethylsulfonyl fluoride
pRb: retinoblastoma protein
scFvs: single-chain variable fragments
SPLINT: single pot library of intracellular antibodies
