# Supplementary material for: A novel intracellular antibody against the E6 oncoprotein impairs growth of human papillomavirus 16-positive tumor cells in mouse models
Source: Oncotarget. 2016 Jan 15;7(13):15539–53. doi: 10.18632/oncotarget.6925 (PMC4941259; doi:10.18632/oncotarget.6925)
Supplement: Supplementary file 1 [file oncotarget-07-15539-s001.pdf]

A novel intracellular antibody against the E6 oncoprotein impairs growth of human papillomavirus 16-positive tumor cells in mouse models

Supplementary Material

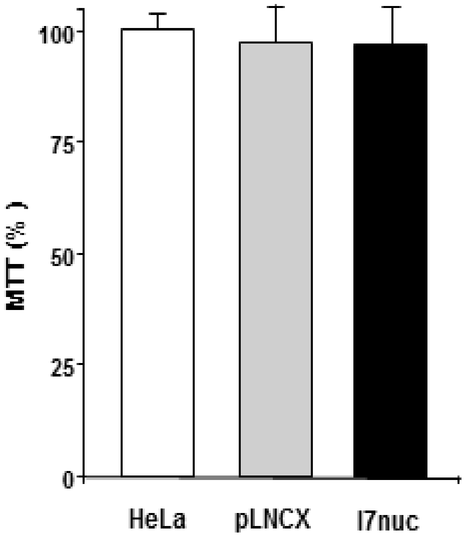

Antiproliferative activity of I7nuc is HPV type specific

HPV18-positive HeLa cells were mock-infected or infected with I7nuc or pLNCX retroviruses. Cell viability was evaluated by MTS assay at 72 h post-infection. Data are expressed as a percentage of MTS conversion in non-transduced cells and represent the mean +/- SD of samples in triplicate from a representative experiment of two with similar results.
